# A Modified Translaminar Osseous Channel-Assisted Percutaneous Endoscopic Lumbar Discectomy for Highly Migrated and Sequestrated Disc Herniations of the Upper Lumbar: Clinical Outcomes, Surgical Indications, and Technical Considerations

**DOI:** 10.1155/2017/3069575

**Published:** 2017-03-30

**Authors:** Zhijun Xin, Wenbo Liao, Jun Ao, Jianpu Qin, Fang Chen, Zhiyuan Ye, Yuqiang Cai

**Affiliations:** Department of Spinal Surgery, The First Affiliated Hospital of Zunyi Medical College, Zunyi, China

## Abstract

Objective is to describe a safe and effective percutaneous endoscopic approach for removal of highly migrated and sequestrated disc herniations of the upper lumbar spine and to report the results, surgical indications, and technical considerations of the new technique. Eleven patients who had highly migrated and sequestrated disc herniations in the upper lumbar were included in this study. A retrospective study was performed for all patients after translaminar osseous channel-assisted PELD was performed. Radiologic findings were investigated, and pre-and postoperative visual analog scale (VAS) assessments for back and leg pain and Oswestry disability index (ODI) evaluations were performed. Surgical outcomes were evaluated under modified MacNab criteria. All of the patients were followed for more than 1 year. The preoperative and postoperative radiologic findings revealed that the decompression of the herniated nucleus pulposus (HNP) was complete. After surgery, the mean VAS scores for back and leg pain immediately improved from 8.64 (range, 7–10) and 8.00 (range, 6–10) to 2.91 (range, 2–4) and 2.27 (range, 1–3), respectively. The mean preoperative ODI was 65.58 (range, 52.2–86), which decreased to 7.51 (range, 1.8–18) at the 12-month postoperative follow-up. The MacNab scores at the final follow-up included nine excellent, one good, and one fair. The modified translaminar osseous channel-assisted PELD could be a safe and effective option for the treatment of highly migrated and sequestrated disc herniations of the upper lumbar.

## 1. Introduction

Upper lumbar disc herniation is disc herniation at the L1-L2, L2-L3, or L3-L4 level, accounting for approximately 5% of all lumbar disc herniations [1]. Due to the special anatomical structure and functional characteristics of the upper lumbar spine, such as the narrow spinal space and the smaller range of motion, the dural sac and nerve structure in the spinal canal of the upper lumbar are more likely to be compressed, which is more likely to manifest as multiple neurological disorders rather than being limited to the involvement of a certain nerve [2]. Therefore, the clinical therapeutic outcome of this type of herniation is often worse than those of the lower lumbar [3]. At the same time, since this disease is difficult to treat conservatively to reach remission, when the diagnosis is clear, surgical treatment is frequently recommended [4]. However, due to the special anatomical and functional characteristics of the high lumbar spine, selection of the appropriate surgical treatment strategy is difficult, especially when the disc herniation is highly migrated and sequestrated (i.e., located beneath the pars interarticularis medial to the pedicle). In these cases, the selection of the surgical method is even more difficult, even for experienced spinal surgeons [5, 6].

Surgical treatments for highly migrated and sequestrated disc herniation of the upper lumbar include the traditional posterior laminectomy and the percutaneous endoscopic technique. However, posterior laminectomy often leads to the destruction of the stability of the motion segments due to its resection of the lamina, the isthmus, and the intervertebral facet joints; further, this procedure can induce postoperative back pain and complications [5–8]. Although the percutaneous endoscopic lumbar discectomy (PELD) technique is recommended for the treatment of highly migrated and sequestrated disc herniations of the upper lumbar region due to its numerous advantages and accelerated patient recovery [9], currently, most PELD techniques are developed from techniques based on Kambin's transforaminal approach or a translaminar approach. These traditional endoscopic approaches have many limitations in the treatment of highly migrated and sequestrated disc herniations of the upper lumbar. Since traditional PELD approaches have the shortcomings of insufficient exposure, inevitable damage to the intervertebral joints, and vision limitation so that the surgeon cannot achieve excision of the herniated nucleus pulposus (HNP), some spinal surgeons suggest that this surgical method is not suitable for the treatment of highly migrated and sequestrated disc herniations of the upper lumbar [4, 5, 10].

To our knowledge, at present, there are few relevant studies on PELD techniques for the treatment of highly migrated and sequestrated disc herniations of the upper lumbar; furthermore, the clinical outcome is also not ideal. Therefore, based on our practical clinical experience in the treatment of highly migrated and sequestrated disc herniations of the upper lumbar using an improved PELD approach, we present a novel technique: translaminar osseous channel-assisted PELD. The present study will elaborate this technique from the aspects of clinical efficacy, indications, and technical references.

## 2. Patients and Methods

### 2.1. Inclusion and Exclusion Criteria

The inclusion criteria were patients with highly migrated and sequestrated disc herniations, which was displaced away from the extrusion site and greater than the posterior marginal disc height measured from the adjacent endplate level of the upper body [4, 5, 10–12], at L1-L2, L2-L3, or L3-L4 level as demonstrated by computed tomography (CT) and magnetic resonance imaging (MRI); single-segment disc herniation; and unsuccessful conservative treatment for at least 6 weeks. The exclusion criteria were the presence of spinal stenosis; clear instabilities or deformities; chronic discogenic back pain; painless motor weakness and pyogenic discitis.

11 patients (7 female, 4 male) who were operated on at our hospital between April 2014 and January 2015 and underwent a translaminar osseous channel-assisted PELD were enrolled. Their ages ranged from 31 to 65 years (mean: 48.1 years). The duration of pain ranged from 53 to 87 days (mean: 68 days). All patients present with back pain and radiating pain to one or both legs.  Table 1 summarizes the clinical characteristics and neuroimaging findings in the studied cases.

### 2.2. Surgical Technique

**Figure 1:**
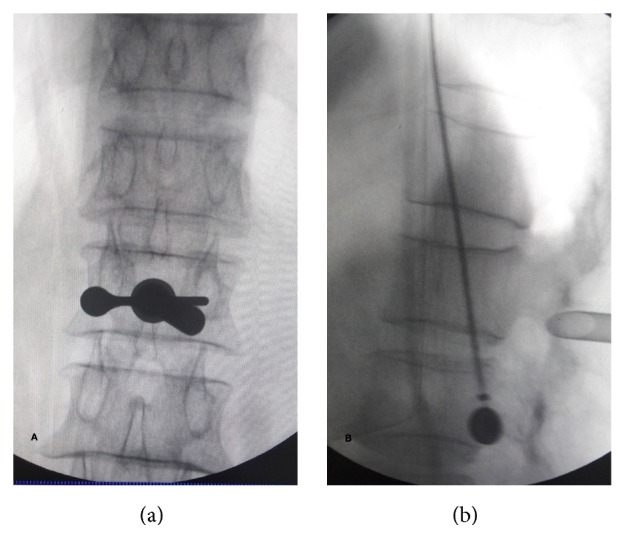
Intraoperative C-arm images of the procedure. Insertion of the working sheath toward the right lower laminar of the lumbar 1 vertebra. Anteroposterior radiograph showing the downward inclination of the working sheath lying at the medial pedicular line (a). Lateral radiograph showing the working sheath piercing the lamina (b).

**Figure 2:**
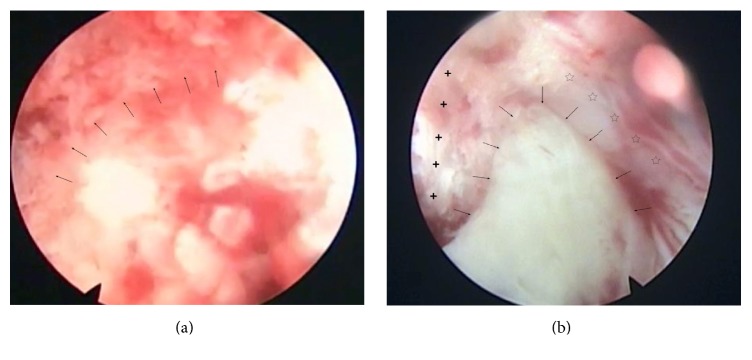
Intraoperative endoscopic view before decompression. A circular osteal groove (black arrows) is made with a trepan on the lamina of the vertebra to site a working sheath (a). The exiting nerve root (stars) is shifted laterally by the disc protrusion fragments (black arrows). The dural sac (crosses) is also compressed by the protrusion fragments, and the protruding fragments (long arrow) can be easily accessed under direct visualization by the endoscope (b).

**Figure 3:**
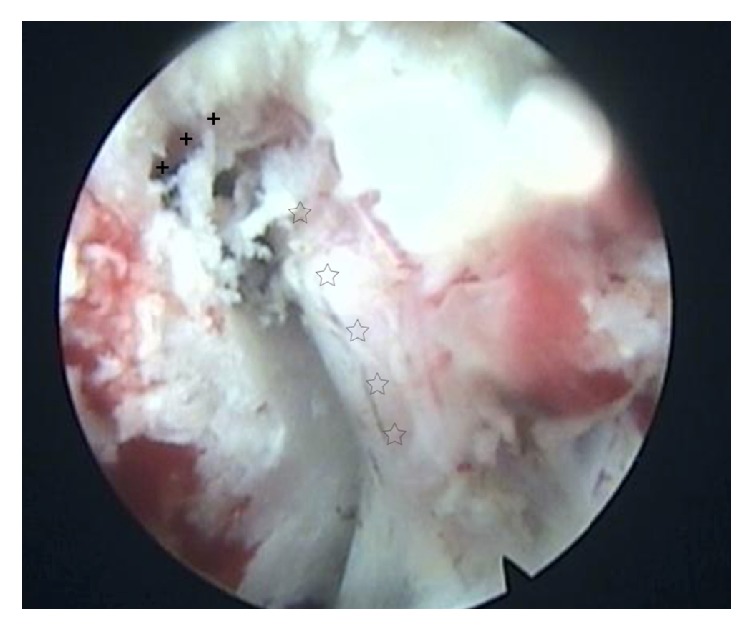
Intraoperative endoscopic view after resection of the protruding fragments; the decompressed dural sac (crosses) and the nerve root (stars) are confirmed, and the protruding fragments are deflected at the axilla of the exiting nerve.

In these cases, a laminar channel was performed in order to assist PELD. The surgeries were under epidural anesthesia and radiographic control with the patient prone by one endoscopic spine surgeon (Liao). Before surgery, we used axial MR or CT images to calculate the distance of skin entry point, and the target point of the needle had been marked on the skin according to preoperative radiological information. Confirming the segment, the line of spinal joints and skin entry point were marked under posterior-anterior radiograph control, positioning skin incision. The needle trajectory aimed at the target position of the laminar, where the HNP lies hidden by the laminar of vertebra, then blunt insertion of a dilator was done with 6.0 mm diameter onto the target position of the laminar. Insertion of the trephine (OD: 7.5 mm, ID: 6.5 mm, Joimax, Germany) was done via the dilator and punching on the laminar, then clockwise rotation and forwarding about 1.0 cm were made, with resecting of the lateral bone of the laminar, and the exacted area of the bone was removed with the trephine. The working sheath used has an inside diameter of 6.5 mm and a beveled opening (Figure 1). Then with insertion of the endoscope through the working canal, further operation is performed under visual control and continuous fluid flow with 0.9% saline solution. Preparation of the ligamentum flavum and the fat in canal, resection of the lateral flavum ligament, and identification of the lateral edge of the dural sac and the exiting root were done. Bipolar radiofrequency coagulation of the venous plexus and preparation of the spinal nerve under particular attention were done. Direct visualization of the HNP can be achieved through the endoscope and intraoperative view revealing the highly migrated and sequestrated fragments located at the axillary region of the exiting root (Figure 2). Remove the HNP using the endoscopic forceps and complete decompression of it (Figure 3). After successful decompression, complete hemostasis was confirmed. After all instruments were removed, direct closure of skin was performed. No drainage was required.

### 2.3. Postoperative Course

The patients had uneventful postoperative recoveries and were discharged at the third postoperative day. MRI and CT scan were done to ensure successful removal of the HNP at the second postoperative day. Visual analog scale (VAS) evaluation for pain and Oswestry disability index (ODI) analysis were repeated in the immediate postoperative period and also at months 3, 6, and 12 after surgery and subsequently if required. A flexion-extension radiograph was also required at the second postoperative day to ensure there was no postoperative instability.

### 2.4. Reference Index

Radiologic findings can be revealed before and after the surgery by the routine lumbar radiographs along with MRI and CT scan.

All patients were evaluated by VAS of 0–10 for back and leg pain with ten being rated as maximum discomfort and assessed clinical function on the basis of ODI score before operative and each postoperative time points. Statistical analysis of the data was performed by SPSS statistical software (version 18.0, Chicago, IL, USA) and repeated measures data of variance were used for the statistical analysis. Relationships between each postoperative time point variable and outcome were analyzed using Bonferroni statistical analysis. Values for *P* less than 0.05 were considered statistically significant.

The surgical outcomes were divided into excellent, good, fair, and poor on the basis of modified MacNab criteria.

## 3. Results

The mean operation time was 82 minutes (range, 65–116). There was no measurable blood loss and the operation was technically feasible in all patients. After the surgery, preoperative symptoms improved in all patients. Postoperative instability was not observed on flexion-extension radiographs. No patient has nerve root injury, cerebrospinal fluid leakage, or major blood vessel and postoperative axial pain at the last follow-up. The mean follow-up period was 16.2 months (range, 14.6–17.1 months).

### 3.1. Radiological Findings

**Figure 4:**
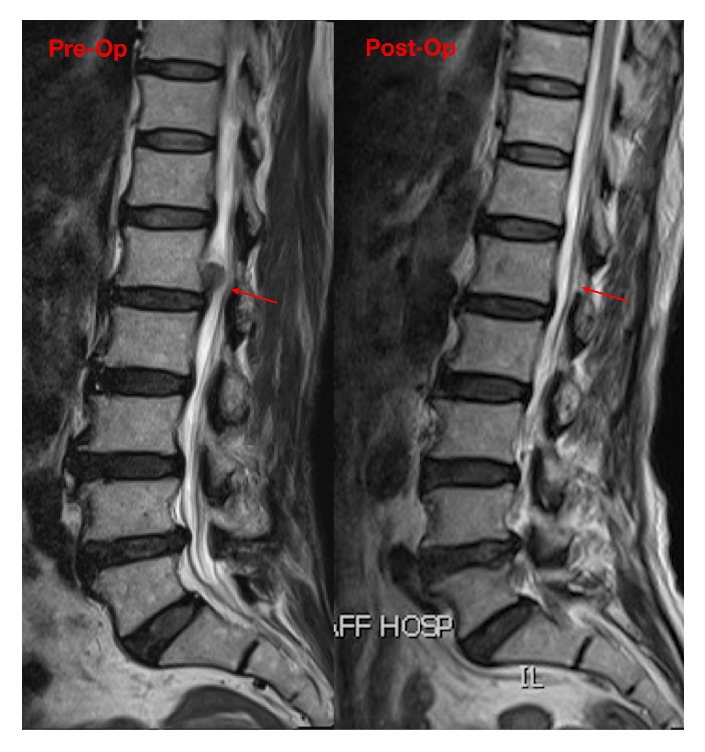
Comparison of magnetic resonance imaging (MRI) findings before and after surgery. Preoperative sagittal plane T2-weighted MRI showing highly upmigrated and sequestrated disc herniation fragments at the L1-L2 level. Postoperative sagittal plane T2-weighted MRI of the same patient demonstrating complete removal of the L1-L2 disc protrusion fragments.

MRI examinations at the second postoperative day demonstrated complete decompression of the HNP and the compressional dural sac and the shifting nerve root were returned (Figure 4).

**Figure 5:**
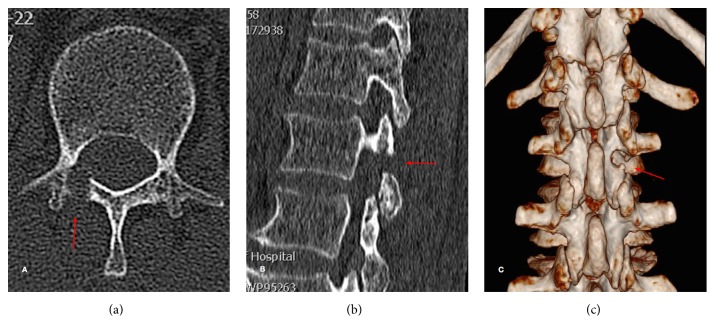
Postoperative computed tomography (CT) scans. The bone resection of the lamina is clearly indicated by CT scans in the axial (a) and sagittal (b) views. Reconstructed CT scans demonstrating no injury on the articular process and the pedicle of the vertebral arch (c).

On the postoperative reconstructed CT scans, the bone resection of the laminar and the translaminar channel is clearly indicated (Figure 5).

### 3.2. VAS for Back and Leg Pain and ODI

The average preoperative VAS scores of back and leg pain and ODI were 8.64 ± 0.28 (range, 7–10), 8.00 ± 0.49 (range, 6–10), and 65.58 ± 3.40 (range, 52.2–86), respectively. Compared with preoperative state, patients had an average VAS of 0.36 ± 0.15 (range, 0-1) and 0.73 ± 0.19 (range, 0–2) for back and leg pain and an average ODI of 7.51 ± 1.45 (range, 1.8–18) at 12 months after surgery.  Table 2 shows the results of the ODI and the VAS scores for back and leg pain; there is a constant and significant (*P* < 0.001) improvement in the pain and activities of daily living. Compared with preoperative state, the ODI and VAS were significantly decreased at each postoperative time point (*P* < 0.05).

### 3.3. Clinical Result

The overall success rate was 90.9%. According to modified MacNab criteria, the final follow-up was excellent in 9 of 11 patients (81.8%) and good in 1 patient (9.1%), and there was only one in fair and none in poor.

## 4. Discussion

Currently, the efficacy of the clinical surgical treatment of sequestrated and migrated disc herniations of the upper lumbar is not ideal. When the disc herniation is located at the upper lumbar level, the selection of the surgical approach is difficult. The particularly special anatomical features of the upper lumbar compared with the lower lumbar, such as the narrow spinal canal, the short and fixed nerve roots, and the narrow lamina window, often lead to inefficacy of surgical treatment for disc herniations of the upper lumbar; selection of the surgical treatment strategy for highly migrated and sequestrated disc herniations of the upper lumbar is even more difficult [5, 6, 13]. Traditional open surgery includes anterior or posterior surgery; however, to achieve the goal of removing HNP, spinal surgeons often need to remove important intervertebral joints or other structures, which often leads to postoperative instability and other complications [8]. In addition, the decompression and fixation of open surgery increase the possibility of poor fusion, bone graft failure, donor site complications, difficult repair, the loss of motion segments, and other issues [14]. Therefore, to reduce the trauma caused by surgery, since Hijikata et al. first reported the percutaneous nucleus pulposus removal technique in 1975 [15], an increasing number of different versions of endoscopic techniques have been reported by spinal surgeons, such as Kambin et al.'s arthroscopic microdiscectomy (AMD) [16], Yeung's selective endoscopic discectomy [17], and Mayer and Brock's PELD technique [18]. Compared with open surgery, PELD techniques provide identical visualization and exposure of the spine, with reduced incidence of operative morbidity and caused less pain, cosmetic benefit, and rapid recovery [9, 19]. However, while some scholars have used endoscopic techniques in the treatment of highly migrated and sequestrated disc herniation of the upper lumbar, others are skeptical about its application [4, 11]. The traditional PELD technique often has two entry approaches: the transforaminal and interlaminar approaches. However, occlusions from osseous obstruction of the pedicle and obstruction of the exiting nerve root and other soft tissues cause limitations in the working area of the transforaminal PELD technique and result in difficulty achieving a complete resection of HNP, especially when treating patients with highly migrated and sequestrated disc herniations of the upper lumbar. Since the space between the exiting root and the articular process is very limited, even when extreme caution is taken, it is impossible to completely avoid the risk of damaging the exiting nerve root [13]. It has been reported that approximately 1.0%–6.7% of patients have exiting nerve root injuries and incomplete HNP removal that necessitate reoperation. However, because of these risks, wide application of the transforaminal PELD technique is limited, especially when the space between the exiting nerve root and the intervertebral joint is narrow [9, 10, 17, 20]. Therefore, for patients with narrow spaces between the nerve root and the facet, some scholars suggest the application of other surgical methods, such as interlaminar PELD. However, for highly migrated and sequestrated disc herniations of the upper lumbar, the relatively narrow interlaminar window and the low interlaminar gap of relatively herniated fragments also limit the implementation of the interlaminar approach PELD; according to Choi's opinion, in particular, the interlaminar PELD technique is only applicable to the L5/S1 level [21]. Some studies in the literature have reported the use of a dural sac surgical approach for the treatment of herniations of the upper lumbar, but this surgical method increases the risk of cerebrospinal fluid leakage, postoperative infection, and nerve injury [22].

Although the application of the PELD technique for the treatment of highly migrated and sequestrated disc herniation of upper lumbar has limitations, due to the numerous advantages of this technique, spinal surgeons have never stopped exploring this technique. Choi et al. [5] and Schubert and Hoogland [23] have reported a modified transforaminal PELD technique, which partially removes the facet or the pedicle to expand the intervertebral foramen to expose the HNP. However, the tip of the articular process can be easily removed using this approach, and because of insufficient visualization of the HNP, it may increase the risk of subsequent open surgery and segmental instability, especially for highly upmigrated and sequestrated HNPs that are hidden from endoscopic view by anatomic barriers like hypertrophied facets, inferior pedicles, and exiting nerve root.

As is generally known, Schellinger et al. [24] reported that most migrated and sequestrated disc herniations are highly migrated and are mainly located in the lower inner side of the pedicle isthmus and break into many pieces. Therefore, the incomplete intraoperative removal of the sequestrated herniated fragments occurs frequently, and its probability in patients with highly migrated and sequestrated herniations is even higher. As reported by Lee et al. [10] and Choi et al. [11], those high-grade migration (HGM) groups (i.e., migration upward or downward beyond the measured height of the posterior marginal disc space) showed even higher rates of surgical failure, the major reason for which lies in the insufficient exposure of the HNP, which causes incomplete removal.

To obtain direct and sufficient exposure of HNP, we used a trephine to make a precise hole on the osseous lamina to establish an osseous channel to assist the PELD. After clinical application, we suggest that in the treatment of highly migrated and sequestrated disc herniations, especially of the upper lumbar, translaminar osseous channel-assisted PELD is a safe and effective supplement to minimally invasive spinal surgical techniques. This procedure has several advantages. ① This minimally invasive surgery can be completed epidurally or under local anesthesia and requires less postoperative care. ② As a fast and accurate manner of channel establishment, precise and targeted translaminar drilling of the hole avoids the likelihood of the occurrence of subsequent instability caused by large removal of articular processes. ③ This surgical method is easy for HNP exposure and can provide an adequate view, including the HNP and the nerve structures, according to the surgeon's need. ④ The most important advantage is that this surgical method reduces the formation of epidural scarring, which is beneficial for the implementation of rerevision operations.

The establishment of an ideal minimally invasive spinal surgical pathway means that it should be directly aimed at the surgical target or as close to the HNP as possible. This consideration is also the key to surgical success, especially in cases of the PELD technique. Our surgical method can meet this requirement. The translaminar osseous channel-assisted PELD, as a novel technique, also has some complications, such as damage to isthmus interarticularis, dural tears, or incomplete removal of the herniated disc. There are many ways to avoid these complications. First, we used preoperative images to calculate the target punching point of the laminar, where it is kept away from the isthmus interarticularis and close to lamina. Second, we used the trephine combined with high-speed diamond drill to punch on the laminar and under the guidance by C-arm X-rays scanning. Third, the scope of the visual control can be widened with an inclined introduction and pivoting motion of the working canal or the endoscope. With this approach, the removal of the HNP can be complete. However, if the HNP is too far away from the working canal to reach to the distal end of the migrated disc, open surgery should be considered.

## 5. Conclusions

However, we do not suggest routine use of this approach for the treatment of unilateral disc herniations of the upper lumbar. Based on our existing experience, we suggest that disc herniation patients of the upper lumbar who meet the following indications can consider using this translaminar osseous channel-assisted PELD technique: ① they have highly migrated and sequestrated disc herniation of the upper lumbar; ② preoperative imaging data confirm that the herniated disc tissue is located at the axillary site of the exiting nerve root and is blocked by the exiting nerve root and the osseous structure of the vertebra; ③ the back of the HNP is blocked by lamina, and the interlaminar PELD surgical method cannot be used for the resection; and ④ no degenerative spinal stenosis, instability, or dislocation is present. Based on existing clinical applications, we suggest that the translaminar osseous channel-assisted PELD surgical method is a safe and effective method for the treatment of highly migrated and sequestrated disc herniations of the upper lumbar. Spinal surgeons should be familiar with this surgical method and its indications.

## Figures and Tables

**Table 1:** Clinical and neuroimaging characteristics in eleven patients with highly migrated and sequestrated disc herniations of the upper lumbar^*∗*^.

Case number	Age (yrs)	Sex	Back pain	Radicular pain	Motor deficit	Sensory deficit	FNST	SLRT	Level of CM	Level of DH
1	65	F	+	rt leg	rt L-1 SD	rt L-1, L-2 SD	+	−	L-1 body	L1-2
2	53	F	+	rt leg	rt L-3 SD	rt L-3 SD	+	+	L1-2 disc	L3-4
3	31	M	+	Both legs	Both L-2 SD	Both L-2 SD	+	−	L-1 body	L2-3
4	45	F	+	lt leg	−	lt L-3 SD	−	−	L-1 body	L3-4
5	37	F	+	rt leg	−	rt L-1, L-2 SD	+	−	L1-2 disc	L1-2
6	43	M	+	Both legs	−	−	−	−	L-1 body	L2-3
7	55	F	+	rt leg	rt L-1 SD	rt L-1 SD	+	−	L-1 body	L1-2
8	47	M	+	Both legs	Both L-2	Both L2 SD	+	−	L-1 body	L1-2
9	32	M	+	lt leg	lt L-3	lt L3, L4 SD	−	+	L-1 body	L3-4
10	59	F	+	Both legs	Both L-2 SD	Both L-2 SD	+	−	L1-2 disc	L2-3
11	62	F	+	lt leg	−	−	+	−	L-1 body	L1-2

^*∗*^No patient had bladder or bowel dysfunction.

CM = conus medullaris; DH = disc herniation; FNST = femoral nerve stretch test; SD = sensory dermatome; SLRT = straight leg raising test; − = absent; + = present.

**Table 2:** Comparison of VAS and ODI in eleven patients before operation and at each postoperative time point ($\overset{-}{x}\pm S$).

	Preoperative	Immediate	3 months	6 months	12 months	*F* values
VAS of back	8.64 ± 0.28	2.91 ± 0.21^*∗*^	2.18 ± 0.12^*∗*#^	1.45 ± 0.16^*∗*#^	0.36 ± 0.15^*∗*#^	*P* < 0.001
VAS of leg	8.00 ± 0.49	2.27 ± 0.24^*∗*^	1.46 ± 0.28^*∗*#^	1.09 ± 0.21^*∗*#^	0.73 ± 0.19^*∗*#^	*P* < 0.001
ODI	65.58 ± 3.40	31.25 ± 2.83^*∗*^	18.93 ± 1.78^*∗*#^	12.16 ± 1.49^*∗*#^	7.51 ± 1.45^*∗*#^	*P* < 0.001

^*∗*^Compared with preoperative, *P* < 0.05.

^#^Compared with immediately after surgery, *P* < 0.05.

## Additional Points

*Device Status/Drug Statement*. This Manuscript submitted does not contain information about medical device(s)/drug(s).
